# Release Kinetic Studies of Aspirin Microcapsules from Ethyl Cellulose, Cellulose Acetate Phthalate and their Mixtures by Emulsion Solvent Evaporation Method

**DOI:** 10.3797/scipharm.0908-09

**Published:** 2009-12-19

**Authors:** Vikas Dash, Sujeet K. Mishra, Manoj Singh, Amit K. Goyal, Goutam Rath

**Affiliations:** 1 Kanak Manjari Institute of Pharmaceutical Sciences, Chhend Colony, Rourkela, Orissa, India; 2 Department of Pharmaceutics, ISF College of Pharmacy, Moga (Punjab), India

**Keywords:** Aspirin, Ethyl Cellulose, Cellulose Acetate Phthalate, Microcapsules, Release Kinetics

## Abstract

The present study was oriented towards microencapsulation of aspirin and the study of its release kinetics. The desired encapsulation was achieved by emulsion solvent evaporation method using ethyl cellulose (EC), cellulose acetate phthalate (CAP) and their mixture (1:1) of polymeric constituents. Characterization of the formulations was performed by size, shape, drug loading efficiency and *in-vitro* drug release analysis. The *in-vitro* release profiles from different polymeric microcapsules were applied on different kinetic models. The prepared microcapsules were found free flowing and almost spherical in shape with particle sizes ranging from 300–700μm, having a loading efficiency of 75–85%. The best fit model with the highest correlation coefficient was observed in Higuchi model, indicating diffusion controlled principle. The *n* value obtained from Korsemeyer-Peppas model varied between 0.5–0.7, confirming that the mechanism of drug release was diffusion controlled. Comparative studies revealed that the release of aspirin from EC microcapsules was slower as compared to that of CAP and their binary mixture.

## Introduction

Acetylsalicylic acid (Aspirin) is a non-steroidal anti-inflammatory and antipyretic drug. It inhibits platelet aggregation and prolongs bleeding time. It is widely used for the relief of pain [1, 2]. It is a Cyclo-oxygenase-2 inhibitor and is amply used as analgesic and anti-inflammatory drug having a half-life of 15–20 minutes [3]. Aspirin has a direct irritant effect on gastric mucosa due to inhibition of prostaglandins and prostacyclins and thus causes ulceration, epigastric distress, and/or hemorrhage [4]. Sustained release formulation of aspirin would reduce the undesired side effects, reduce frequency of administration and improves patient compliance [5].

Encapsulation is a useful method for prolonging the drug release from dosage forms and reducing adverse effects [6–8].

Microcapsules are composed of a polymer wall enclosing a liquid core or other body of material [9]. Microencapsulation can be achieved by a myriad of techniques. The emulsion solvent evaporation (ESE) method can be used to prepare microcapsules of water insoluble drug [10–13]. Moreover, addition of non-solvent also markedly affects the morphological and release kinetics of resultant particles [14, 15].

In this present study, Aspirin microcapsules were prepared by emulsion solvent evaporation technique using EC, CAP and their combination as a coating material [16, 17]. Ethanol and acetone were used for the preparation of microcapsules. The prepared microcapsules were evaluated for drug content, particle-size, SEM and for in-vitro drug release study.

## Results and Discussion

Aspirin microcapsules were prepared by emulsion solvent evaporation (ESE) methods using ingredients as given in Table 1.

### Morphology

**Fig. 1.**
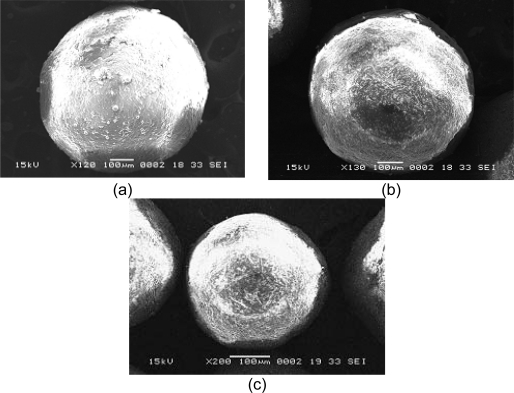
Scanning Electron Microscopy of aspirin microcapsules with polymers: (a) CAP, (b) EC and (c) EC+CAP

The developed microcapsules were found to be discrete, spherical, free flowing and white in colour. SEM was performed on the developed microcapsules to assess their surface and morphological characteristics as shown in figure 1. SEM studies revealed that the microcapsules are almost spherical in shape.

### Particle Size

Particle size of developed formulations was determined by particle size analyzer (Mastersizer Hydro 2000MU). Particle size analyses of various formulations are presented in table 2. Studies revealed that EC based microcapsules were larger than CAP, and EC + CAP based microcapsules. This may be attributed to differences in evaporation rate and solubility index of polymers in respective solvents.

### Drug entrapment efficiency

The yield of microcapsules was found in the range of 90–94 %. The drug content was found to be higher in CAP microcapsules followed by EC and CAP+EC as depicted in (Table 2). The higher drug entrapment in CAP microcapsules was attributed to the percentage yield, nature and concentration of polymer in the internal phase.

### In-vitro drug release

**Fig. 2.**
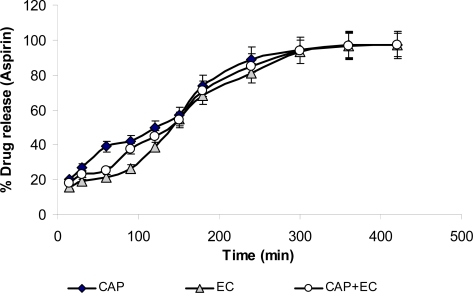
Comparative Release Profile of aspirin Microcapsules with different polymers (EC, CAP and EC+CAP)

Aspirin release from the microcapsules was studied at pH-1.2 for 2 hrs followed by in acetate buffer at pH 6.0 for 7 hrs. The release profiles of microcapsules are graphically presented in Figure 2. Result revealed that the *In-vitro* drug release of aspirin at pH-1.2 for first two hours was very slow followed by instant release at pH-6.0. After seven hours of dissolution, the amount of drug release from the microcapsules containing EC as coating material was found to be the least, whereas in case of CAP microcapsule, the release of aspirin after two hours was maximum. Drug release from all the microcapsules followed first order kinetics. The order of aspirin release from the microcapsules containing various polymers followed CAP > CAP + EC > EC. The release pattern indicated that the drug was released at constant rate and controlled by more than one mechanism.

Release of aspirin from all the polymeric microcapsules were slow and spread over longer period of time. The release of aspirin at pH-1.2 was very slow which could be due to poor solubility of the drug and polymer in acidic medium. After 2 hrs when the microcapsules were transferred to acetate buffer at pH-6.0, there was abrupt release of the drug indicating that the medium can diffuse into polymeric matrix and dissolve the drug.

### Release Kinetics

The release kinetics of aspirin from various formulations was determined by comparing their respective co-relation coefficients. aspirin release from the microcapsules followed first-order kinetics. The plot of log % drug remaining vs. time was found to be linear with correlation coefficient greater than that of the zero order kinetics (Table 3).

Table 3 shows the correlation coefficient of different kinetic models for aspirin microcapsules containing various polymers. Higuchi plots (Table 3) were found to be of highest linearity with correlation coefficient greater than that of the zero order kinetics and corresponded to that of the first order kinetics indicating that the drug release mechanism from these microcapsules was diffusion controlled and follow first order kinetics in simulated intestinal fluids. Studies revealed that release of aspirin from developed microcapsules was found to be very close to zero-order kinetics in simulated gastric fluid, indicating that the concentration was nearly independent of drug release. Moreover, *in-vitro* release of aspirin from CAP microcapsules was best explained by Korsmeyer-Peppas equation indicated a good linearity (r^2^=0.978). The release exponent n was 0.514, which indicates a coupling of the diffusion and erosion mechanism so called anomalous diffusion and may indicate that the drug release is controlled by more than one process. Furthermore, other formulations were also explained by Korsmeyer-Peppas equation and indicated that value of “n” in Peppas equation is 0.5 < n < 1.0, which implies that the drug follows non- Fickian transport.

The results indicated that EC and CAP combination based formulation exhibited the slowest release rate in SGF followed by faster release in SIF. This may be due to the presence of ethyl cellulose on the surface of microcapsules during their formation. During the formation microcapsules with combination of EC and CAP, acetone evaporated very rapidly compared to ethanol, this may be the contributing factor for the coating of EC over CAP. Release kinetic studies also revealed that plain EC and combination of EC and CAP follow almost similar patterns.

It can be concluded that emulsification/solvent evaporation technique is a reproducible and simple method for the preparation of aspirin microcapsules. It was found that the prepared microcapsules were spherical, free flowing, high percentage entrapment efficiency and high percentage yielding capacity. It can be concluded from this study that aspirin microcapsules could be made suitable for oral controlled drug delivery systems using cellulose acetate phthalate and ethyl cellulose as retardant materials. However, the *in-vitro* release characteristics of drug from the microcapsules are subject to confirmation in animal and human studies for concluding enhanced bioavailability and reduced dose frequency for improved patient compliance.

## Experimental

### Materials

Aspirin (Advent Research Centre, Mumbai), acetone, cyclohexane, hydrochloric acid, ammonium acetate, glacial acetic acid were purchased from E-Merck Ltd., Mumbai. Ethanol, Heavy liquid paraffin, ethyl cellulose, cellulose acetate phthalate and deionized water were purchased from Cosmochem, Pune. All chemicals used in the experiments were of analytical grade and purchased from their respective commercial sources.

### Method

Microcapsules of ethyl cellulose containing aspirin were prepared by an emulsion-solvent evaporation method as reported by Nokhodchi and Farid, 2002 [18]. Aspirin microcapsules were prepared by dissolving polymers in an organic solvent to form a homogeneous polymer solution. The core material, aspirin was added in a thin stream of heavy liquid paraffin. The mixture was agitated using a propeller mixer with the rotation speed 600rpm.The dispersed phase consisting of drug and polymer (CAP, EC and their binary mixtures) were immediately transformed into fine droplets, which subsequently solidified into rigid microcapsules due to solvent evaporation. The liquid paraffin was decanted, and the microcapsules were collected, washed twice in cyclohexane to remove any adhering oily phase (liquid paraffin), and was air dried for at least 12 h to obtain discrete microcapsules. The quantity of polymer, drug and the solvent used is given in Table 1.

### Scanning Electron Microscopy (SEM) studies

The microcapsules were observed under scanning electron microscopy (JEOL, JSM-6840LV, Japan). For SEM the microcapsules were mounted directly on SEM stub, using double sided sticking tape and coated with platinum under reduced pressure and then placed in scanning electron microscope. Photomicrographs of prepared formulations are shown in Figure 1.

### Particle size analysis

The microcapsules were sieved by the standard sieves and arranged in such a manner that the coarsest remained at the top and finest at the bottom. After that the samples retained at the bottom were collected. These samples were determined by photon correlation spectroscopy using Malvern Zetasizer (Mastersizer Hydro 2000MU). The average of 22 measurements was used. All measurements were made at a constant scattering angle of 90° at 25°C. All the data analysis was performed in automatic mode. The results are recorded in Table 2.

### Determination of drug content, encapsulation efficiency and % yield in the microcapsules

Microcapsules (100 mg) of aspirin were weighed and dissolved in 1000 ml 1N HCl by stirring for 30 minutes. The solution was filtered and the filtrate was analysed by appropriate dilution and used for determination of drug entrapment efficiency using the following relationship:

$$\text{Encapsulation}\;\text{efficiency}=\frac{\text{estimated}\;\text{percent}\;\text{drug}\;\text{content}}{\text{theoretical}\;\text{percent}\;\text{drug}\;\text{content}}\times 100$$

The percentage yield of the microcapsules was determined for drug and was calculated using the following equation:

$$\text{Yield}=\frac{\text{M}}{{\text{M}}_{\text{o}}}\times 100$$

Where M is the weight of microcapsules and Mo is the total expected weight of drug and polymer. Drug content, encapsulation efficiency and yield for each formulation were reported in Table-2.

### In-vitro drug release studies

Release of aspirin from the microcapsules was studied at 1.2 pH (900 ml) for the first 2 hrs and followed by in acetate buffer atF pH 6.0 (900 ml) upto 5 hrs using an USP XXIV single stage Dissolution Test Apparatus with a paddle stirrer at 50 rpm. 100 mg of microcapsules was properly weighed and taken in a pouch tied to a glass slide and was placed in 900 ml of dissolution fluid (pH-1.2) and maintained at 37 ± 0.2 ºC. At appropriate intervals (15, 30, 60, 90, 120 mins), 5 ml of the sample was taken and filtered through 0.45 μm Millipore. The dissolution media was then replaced by 5 ml of fresh dissolution fluid to maintain a constant volume. After two hours, the pouch containing microcapsules was removed and placed in 900 ml of dissolution fluid containing acetate buffer (pH-6.0) and then samples were collected at an appropriate intervals (15, 30, 60, 120, 180, 240 and 300 mins) as mentioned above. The samples were then analyzed at 278.5 and 239.5 nm by Elico UV-Visible Double beam Spectrophotometer at pH-1.2 and 6.0, respectively. In order to investigate the mode of release from microcapsules, the release data were analyzed using following mathematical models: Zero-order kinetic (equation 1); first-order kinetic (equation 2); Higuchi equation (square-root of time equation, equation 3) [19]; and Peppas equation (equation 4) [20].

Eq. 1. $$\text{Q}={\text{k}}_{\text{o}}\text{t}$$

Eq. 2. $$\text{ln}(100-\text{Q})=\text{ln}({\text{Q}}_{\text{o}})-{\text{k}}_{1}\text{t}$$

Eq. 3. $$\text{Q}={\text{k}}_{\text{H}}{\text{t}}^{1/2}$$

Eq. 4. $$\text{Log}\;(\text{Q}/100)={\text{k}}_{\text{P}}{\text{t}}^{\text{n}}$$

In equations Q, the percent of drug released is at time t, Q_0,_ the percent of drug remaining to release and k_0_, k_1_ and k_H_ are the coefficients of the equations. k_P_ is constant incorporating structural and geometric characteristics of the release device, and n is the release exponent indicative of the mechanism of release.

## Figures and Tables

**Tab.1.** Quantity of polymer, drug and the solvent

**Formulation**	**Drug: Polymer**	**Liquid Paraffin (ml)**	**Acetone (ml)**	**Ethanol (ml)**	**CAP (gm)**	**EC (gm)**
Aspirin:CAP	4:1	60	10	–	1	–
Aspirin:EC	4:1	60	–	10	–	1
Aspirin:CAP+EC	4:1	60	5	5	0.5	0.5

N=3

**Tab.2.** Particle size, % yield, Drug content and Entrapment efficiency of aspirin Microcapsules

**Property**	**CAP**	**EC**	**CAP + EC**
Particle size (μm)	378.05±30.4	389.02±25.4	210.24±26.6
% Yield	93.86±2.4	91.78±4.5	90.80±3.5
Drug content(gm)	3.418±0.1	3.233±0.15	3.087±0.18
% Entrapment efficiency	85.45±4.3	80.82±5.2	77.17±3.2

N=3

**Tab. 3.** Correlation coefficient (R^2^) and constant (K) of different kinetic models for aspirin microcapsules

**Microcapsule**	**Zero order**		**First order**		**Higuchi equation**		**Peppas equation**		
	**R^2^**	**K_0_**	**R^2^**	**K_1_**	**R^2^**	**K_H_**	**R^2^**	**Kp**	**n**

CAP	0.899	0.225	0.969	0.004	0.974	5.19	0.978	2.61	0.514
EC	0.935	0.245	0.955	0.004	0.944	5.45	0.922	3.47	0.644
CAP & EC	0.924	0.236	0.964	0.004	0.963	5.34	0.953	3.01	0.581
